# Synergizing multimodal data and fingerprint space exploration for mechanism of action prediction

**DOI:** 10.1093/bioinformatics/btaf223

**Published:** 2025-06-03

**Authors:** Kaimiao Hu, Jianguo Wei, Changming Sun, Jie Geng, Leyi Wei, Qi Dai, Ran Su

**Affiliations:** College of Intelligence and Computing, Tianjin University, Tianjin, 300072, China; College of Intelligence and Computing, Tianjin University, Tianjin, 300072, China; CSIRO Data61, Sydney, 2000, Australia; Department of Cardiology, Chest Hospital, Tianjin University, Tianjin, 300041, China; School of Information, Xiamen University, Xiamen, 361005, China; Centre for Artificial Intelligence Driven Drug Discovery, Faculty of Applied Science, Macao Polytechnic University, Macao SAR, 999078, China; College of Life Science and Medicine, Zhejiang Sci-Tech University, Hangzhou, 310023, China; College of Intelligence and Computing, Tianjin University, Tianjin, 300072, China

## Abstract

**Motivation:**

Effective computational methods for predicting the mechanism of action (MoA) of compounds are essential in drug discovery. Current MoA prediction models mainly utilize the structural information of compounds. However, high-throughput screening technologies have generated more targeted cell perturbation data for MoA prediction, a factor frequently disregarded by the majority of current approaches. Moreover, exploring the commonalities and specificities among different fingerprint representations remains challenging.

**Results:**

In this paper, we propose IFMoAP, a model integrating cell perturbation image and fingerprint data for MoA prediction. Firstly, we modify the Res-Net to accommodate the feature extraction of five-channel cell perturbation images and establish a granularity-level attention mechanism to combine coarse- and fine-grained features. To learn both common and specific fingerprint features, we introduce an FP-CS module, projecting four fingerprint embeddings into distinct spaces and incorporating two loss functions for effective learning. Finally, we construct two independent classifiers based on image and fingerprint features for prediction and for weighting the two prediction scores. Experimental results demonstrate that our model achieves highest accuracy of 0.941 when using multimodal data. The comparison with other methods and explorations further highlights the superiority of our proposed model and the complementary characteristics of multimodal data.

**Availability and implementation:**

The source code is available at https://github.com/ s1mplehu/IFMoAP. The raw image data of Cell Painting can be accessed from Figshare (https://doi.org/10.17044/scilifelab.21378906).

## 1 Introduction

Mechanism of action (MoA) refers to the interaction between a compound and molecules, cells, or physiological processes within a biological system, elucidating how it induces specific biological effects or pharmacological responses (Swinney 2004). Exploring the MoA of compounds not only provides guidance for drug design but also enables the prediction of drug efficacy and side effects (Bauer *et al.* 2018, Kiriiri *et al.* 2020). Therefore, accurate prediction of the MoA of compounds is crucial for drug development and drug therapy.

Traditionally, the investigation of the MoA of compounds involves wet-lab experimentation, primarily encompassing animal model studies. The studies involve the administration of compounds to animals via oral, injection, or other routes, followed by the observation of their biological effects within the animal’s body (Mukherjee *et al.* 2022). This enables the assessment of the mechanisms of action within the entire biological system. Drawbacks of animal model studies involve limitations due to ethical and moral concerns. An early computational method on molecular docking infers MoA by calculating the binding mode and affinity of simulated compounds with target proteins (Morris and Lim-Wilby 2008). However, this method is limited by the unknown 3D structures of most proteins (Moreira *et al.* 2010). Another mainstream method is the ligand based method, which is based on the structural information of ligands or compounds for analysis and prediction, mainly including quantitative structure-activity relationship (QSAR) and molecular similarity analysis (Sliwoski *et al.* 2014, Vázquez *et al.* 2020). QSAR extracts molecular descriptors representing compound properties, and then establish models such as support vector machine, random forest and linear regression to predict specific properties of compounds (Svetnik *et al.* 2003, Doucet *et al.* 2007, Polishchuk *et al.* 2009, Tropsha *et al.* 2024). Most of the above methods only focus on structural information and cannot capture complex topological information between atoms. Researchers represent compounds as a graph structure (atoms are nodes and chemical bonds are edges) and apply graph neural network-based models to learn molecular representations(Khemani *et al.* 2024). Lv *et al.* constructed a meta learning framework with graph attention networks (GAT), which extracted the interactions between atomic pairs and edge features of bonds in molecules, achieving good performance in tasks such as toxicity prediction (Lv *et al.* 2024). Yang *et al.* proposed GENNDTI which introduces router nodes based on biological knowledge to construct message-passing paths, utilizing graph convolutional neural networks (GCN) to extract features of the enhanced graph for prediction (Yang *et al.* 2024). Molecular similarity analysis is based on the principle of “guilt by association,” leveraging similarities between molecules to infer properties or functions (Chen *et al.* 2024). Luo *et al.* integrated multi-source data on drugs and targets, computed similarity matrices for various drugs and targets, and utilized them as features to predict drug-target interactions (Luo *et al.* 2017). The existence of an “activity cliff” phenomenon among structurally similar compounds indicates the limited reliability of predictions based solely on structural information, necessitating the integration of more targeted data (Bajorath 2017).

The application of high-throughput screening technology in large-scale compound libraries can effectively and rapidly screen compounds, systematically evaluating their biological activity(An and Tolliday 2010). Correspondingly, Cell Painting, a high-throughput cell imaging technique, has constructed a biological activity measurement database by capturing targeted information on cell morphology and subcellular structure, providing an effective tool and platform for a deeper understanding of biological activity (Bray *et al.* 2016, 2017). Caicedo *et al.* profiled a set of lung adenocarcinoma-associated somatic variants using Cell Painting, and utilized cell morphology analysis to predict the functional impact of the variants (Caicedo *et al.* 2022). Tian *et al.* extracted features from molecule fingerprint and cell morphology to predict MoA (Tian *et al.* 2023). Although these works apply information related to cell morphology, they ignore or only consider single molecular fingerprint, and information is compensated between multiple types of molecular fingerprints. Therefore, there is still room for improvement in these methods.

In this study, we proposed a model called IFMoAP to evaluate the influence of perturbed cell images and multi types of molecular fingerprints for MoA prediction. The main contributions are as follows:

- For the multi-channel cell perturbation images, we borrowed the idea of depthwise separable convolution based on Res-Net50 (He *et al.* 2016), modifying the depthwise convolution into dilated convolution to capture multi-scale features of cells. A granularity-level attention mechanism is proposed to integrate coarse-grained features and fine-grained features, because coarse-grained features are also significant for cells.
- We collected four distinct types of molecular fingerprints for compounds to enhance their representation diversity. Different definitions of fingerprint representations should possess their own specificity, while multiple representations pertaining to the same compound should exhibit commonalities in a certain space. To extract common and specific features of fingerprints, we designed two encoders and proposed two loss functions to enable effective learning of fingerprint features.
- The models corresponding to the two modalities of data were trained independently, with prediction results surpassing those of the baseline methods within the same modality. Our model achieved peak performance when fed with multimodal data, indicating the complementary nature of cell perturbation image data and structural data, which benefits MoA prediction.

## 2 Materials and methods

**Figure 1.**
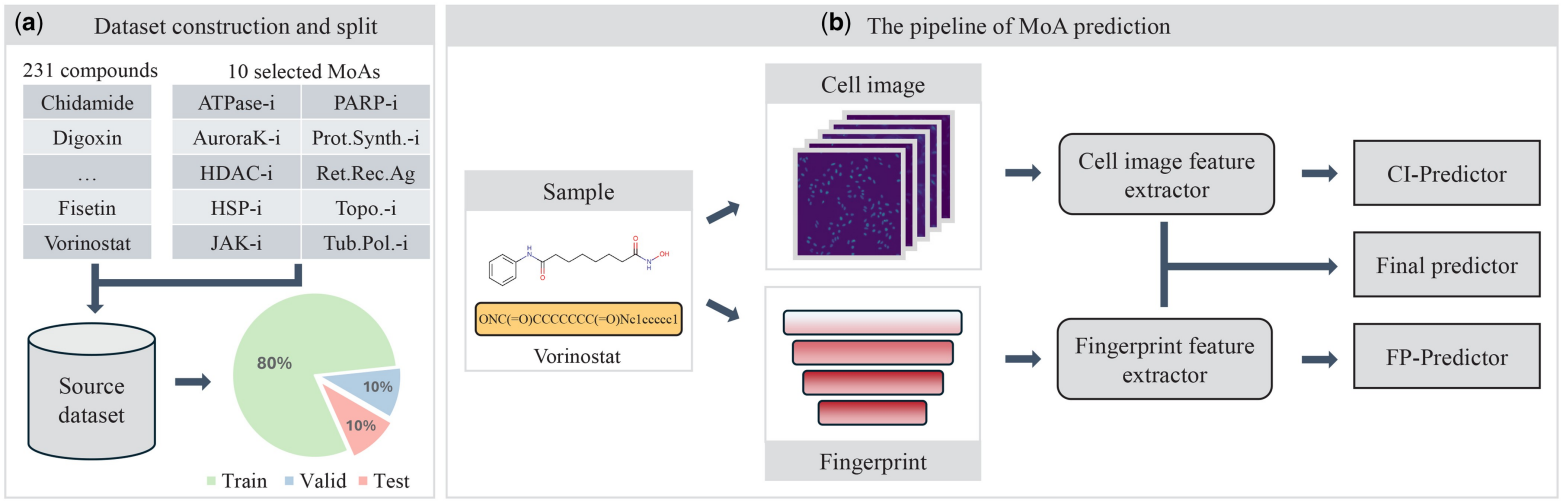
Our proposed overall framework for MoA prediction, including (a) dataset construction and split, and (b) the pipeline of MoA prediction.

In this section, we first introduce the experimental data, including its composition and data matching. Then, we describe the proposed feature extractor for two modalities of data, and finally we provide the predictor and model training loss functions. Figure 1 shows the overall framework used for MoA prediction.

### 2.1 Dataset and data matching

We obtain an experimental dataset from the work of Tian *et al.* (2023) and collected a total of 231 compounds for 10 representative MoA. Tian *et al.* applied the Cell Painting technique to capture cell images from the multi-well plates, acquiring five-channel cell images. The images contain information on cell morphology, subcellular structure, and state. In addition, the simplified molecular input line entry system (SMILES) is a string-based notation used for representing chemical structures. We apply the RDKit library to parse and process compounds represented in the SMILES format, converting them into four types of molecular fingerprints: RDK fingerprint, ECFP fingerprint, PubChem fingerprint, and MACCS fingerprint.

Suppose there are *N* compounds, defined as $\left\{c_{1},c_{2},\ldots ,c_{N}\right\}$, each compound sample has two types of original data representations. For example, the *i*th compound is represented by image representation $c_{i}^{im}$ and fingerprint representation $c_{i}^{fp}$. The five channel cellular image $c_{i}^{im}$ is represented as $\left[{\text{IM}}_{i}^{1}|{\text{IM}}_{i}^{2}|{\text{IM}}_{i}^{3}|{\text{IM}}_{i}^{4}|{\text{IM}}_{i}^{5}\right]$. The fingerprint representation $c_{i}^{fp}$, consists of a collection of four fingerprint vectors: $\left\{{\text{FP}}_{i}^{R},{\text{FP}}_{i}^{E},{\text{FP}}_{i}^{P},{\text{FP}}_{i}^{M}\right\}$. The same compound can be matched with multiple images, while the fingerprint vector remains constant. Each sample after data matching contains a five-channel image and four fingerprint vectors. Further details on the datasets and data preprocessing can be found in Supplementary Section S1.

### 2.2 Multimodal feature extraction

To address the inherent differences in properties and information content in multimodal data of compounds, we develop separate feature extractors to extract image and fingerprint features separately. Specificly, we use the modified Res-Net (MRes-Net) as a cell image feature extractor (CI-Extractor), which excels in extracting salient features from cell images. A fingerprint feature extractor (FP-Extractor) called FP-CS is proposed to identify the specificity and commonality of multi-type fingerprint representations. We use the compound $c_{i}$ as an example to provide a detailed introduction of two feature extractors.

#### 2.2.1 CI-Extractor

Cell images collected through the Cell Painting technique offer a detailed display of cell morphology following compound perturbation, presenting a comprehensive and visual insight into cellular responses. We choose ResNet-50 as the backbone network for extracting cell image features. ResNet-50 is viewed as consisting of 5 stages, defined as [STAGE 0→STAGE 1→STAGE 2→STAGE 3→STAGE 4]. As the initial component of ResNet, the function of STAGE 0 can be regarded as preprocessing the input image. However, due to the unique characteristics of cell images, which have five channels instead of the three channels found in natural images, and the presence of multiple scales within cellular structures, we make modifications to the STAGE 0 of ResNet.

**Figure 2.**
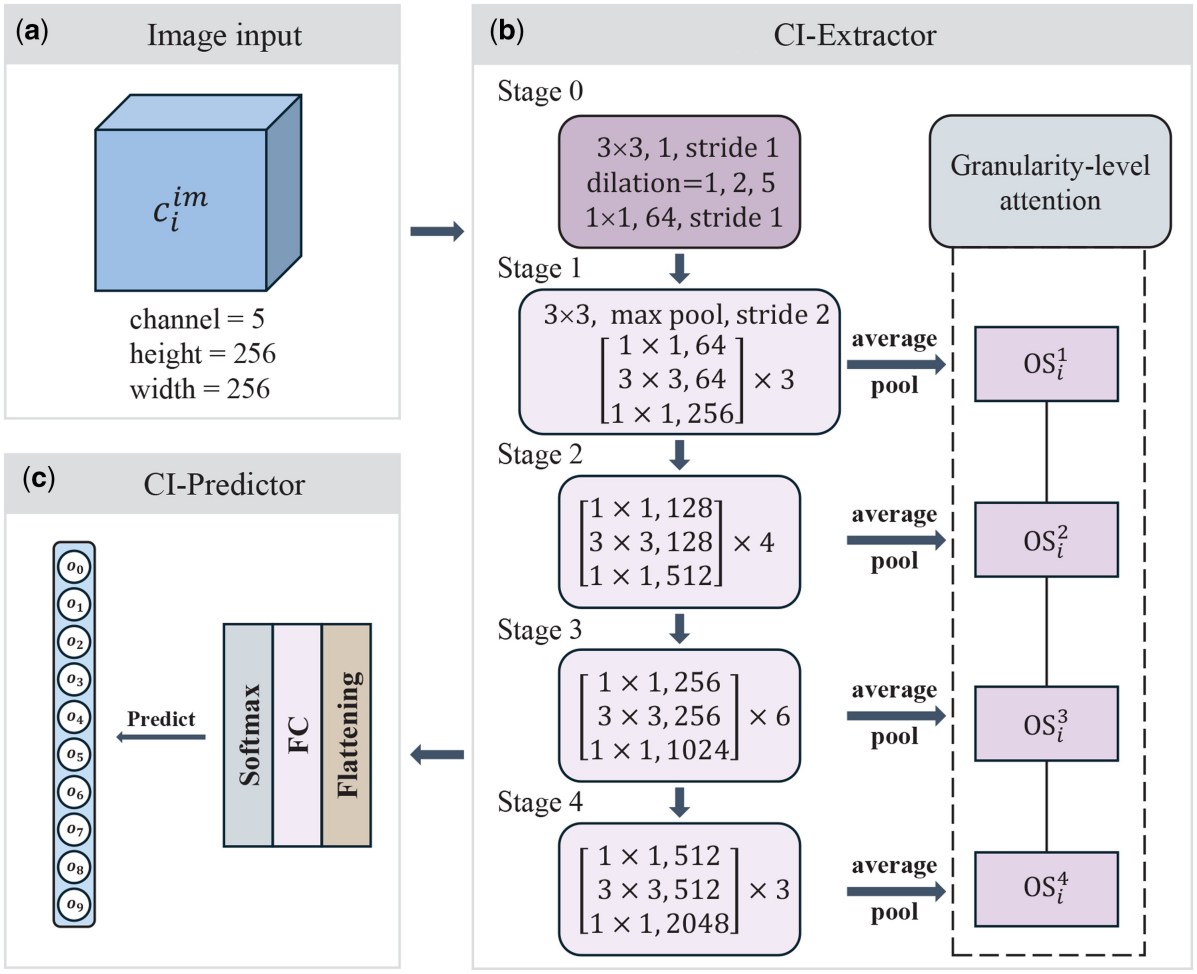
One branch for perturbing cell images to predict MoA, including (a) image input, the format of cell images, (b) CI-Extractor, a cell image feature extractor, and (c) CI-Predictor, an independent MoA classification predictor for extracted cell image features, where $o_{0},\ldots ,o_{9}$ represent the predicted probabilities for the 10 MoA categories.

As shown in Fig. 2b, these modifications incorporate dilated convolutions and depthwise separable convolutions to obtain multi-scale features. The previous work using Res-Net as image feature extraction only focused on the deep features (fine-grained features) output by STAGE 4, which is more advantageous for tasks such as natural image semantic recognition. However, the texture, edge, and geometry information that shallow features (coarse-grained features) focus on are still important for cell images. We defined ${\text{OS}}_{i}^{k}\;(k\in [1,2,3,4])$ is the average pooled feature of the output of the STAGE *k* module after passing through the average pooling layer, and cascaded these features of the remaining stages after STAGE 0,


(1) $${\text{OS}}_{i}=\left[{\text{OS}}_{i}^{1}|{\text{OS}}_{i}^{2}|{\text{OS}}_{i}^{3}|{\text{OS}}_{i}^{4}\right].$$

To better integrate cross-granularity features, we established an attention mechanism at the granularity-level,


(2) $$\alpha =f(W\cdot {\text{OS}}_{i}+b),$$

(3) $$F_{i}^{\text{CI}}=\alpha ⊙({\text{OS}}_{i}),$$

where α can be regarded as the attention score for each feature calculated based on cross-granularity features, b is a bias vector, and ⊙ is element-wise product. $F_{i}^{\text{CI}}$ is the ultimate cell image feature, encompassing morphological characteristics of cellular organelles and information at multiple granularities.

#### 2.2.2 FP-Extractor

Each compound can be represented by four types of molecular fingerprints, each of which is defined based on different concepts. These fingerprint data contain both complementary and redundant information. Therefore, the FP-CS module is proposed, including a fingerprint common encoder (FPC) and a fingerprint specific encoder (FPS), to extract common and specific features from fingerprints.

First, the fingerprint vectors ${\text{FP}}_{i}^{R}$, ${\text{FP}}_{i}^{E}$, ${\text{FP}}_{i}^{P}$, and ${\text{FP}}_{i}^{M}$ of compound $c_{i}$ are transformed with independent affine transformations, resulting in four fingerprint embeddings,


(4) $$\begin{array}{cc} & {\text{fp}}_{i}^{R}=\text{Linear}(W^{R}\cdot {\text{FP}}_{i}^{R}+b^{R}),{\text{fp}}_{i}^{E}=\text{Linear}(W^{E}\cdot {\text{FP}}_{i}^{E}+b^{E}),\\ & {\text{fp}}_{i}^{P}=\text{Linear}(W^{P}\cdot {\text{FP}}_{i}^{P}+b^{P}),{\text{fp}}_{i}^{M}=\text{Linear}(W^{M}\cdot {\text{FP}}_{i}^{M}+b^{M}), \end{array}$$

and they are projected and distributed in their respective semantic spaces.

*FPC encoder and loss:* A compound can be represented by four types of molecular fingerprints, indicating that the corresponding fingerprint embeddings should also be distributed at the same location within the common space of the compound. To capture these inherent common features, we developed an FPC encoder and a loss function. Although ${\text{fp}}_{i}^{R}$, ${\text{fp}}_{i}^{E}$, ${\text{fp}}_{i}^{P}$, and ${\text{fp}}_{i}^{M}$ have the same dimensionality after affine transformation, their semantics are distinct. To ensure that the embedding dimensions and their meanings are consistent, we add zeros to the embeddings, such as ${\text{fp}}_{i}^{R}$ are defined as $\left[{\text{fp}}_{i}^{R},0,0,0\right]$. The vector with zero-padding is still denoted by the original symbol. FPC is a fully connected neural network encoder consisting of two linear layers with activation functions. The padded fingerprint embeddings are input into the FPC, which maps them to the common space,


(5) $$\begin{array}{cc} & C_{i}^{R}=\text{FPC}({\text{fp}}_{i}^{R}),C_{i}^{E}=\text{FPC}({\text{fp}}_{i}^{E}),\\ & C_{i}^{P}=\text{FPC}({\text{fp}}_{i}^{P}),C_{i}^{M}=\text{FPC}({\text{fp}}_{i}^{M}). \end{array}$$

In the common space, the initial four encoded embeddings are scattered at different positions. The target embedding $C_{i}^{T}$ is constructed to align four encoded embeddings, and its value is the mean of their feature values, defined as


(6) $$C_{i}^{T}=(C_{i}^{R}+C_{i}^{E}+C_{i}^{P}+C_{i}^{M})/4.$$

To encourage $C_{i}^{R}$, $C_{i}^{E}$, $C_{i}^{P}$, and $C_{i}^{M}$ to approach $C_{i}^{T}$ in the common space and to make the values of the four encoded embeddings similar, we construct a loss function $L_{c}$,


(7) $$L_{c}={\left\|C_{i}^{R}-C_{i}^{T}\right\|}^{2}+{\left\|C_{i}^{E}-C_{i}^{T}\right\|}^{2}+{\left\|C_{i}^{P}-C_{i}^{T}\right\|}^{2}+{\left\|C_{i}^{M}-C_{i}^{T}\right\|}^{2}.$$

We define the sum of four encoding embeddings after learning, $C_{i}$, as the common feature of the *i*th compound.

*FPS encoder and loss:* The four types of molecular fingerprints consider molecular characteristics from different perspectives, thus should possess specificity. In order to extract specific features of fingerprint embedding, we construct the FPS encoder. The FPS consists of four independent fully connected encoders, and each fully connected encoder is composed of two linear-ReLU layers. The ${\text{fp}}_{i}^{R}$, ${\text{fp}}_{i}^{E}$, ${\text{fp}}_{i}^{P}$, and ${\text{fp}}_{i}^{M}$ are mapped to the specific space through their corresponding fully connected encoders, and we can obtain four encoder embeddings, $S_{i}^{R}$, $S_{i}^{E}$, $S_{i}^{P}$, and $S_{i}^{M}$. To extract unique information from diverse fingerprints and learn independent specific features, we design a new loss function, denoted as $L_{s}$. We concatenate along the first dimension of the four embeddings to obtain $H_{i}$, and then $H_{i}$ is multiplied with its transpose to obtain the correlation matrix between the embeddings,


(8) $$I_{cm}=H_{i}\times {\left(H_{i}\right)}^{T},I_{cm}\in R^{4\times 4},$$

$L_{s}$ is defined as


(9) $$L_{s}={\left\|I_{cm}-I\right\|}^{2},$$

where $I\in R^{4\times 4}$ is the identity matrix, and minimizing $L_{s}$ encourages the encoded embeddings to be more independent. Due to the independence of the four embeddings, we opt to concatenate them together and define specific feature as $S_{i}$,


(10) $$S_{i}=\left[S_{i}^{R}|S_{i}^{E}|S_{i}^{P}|S_{i}^{M}\right].$$

*Integrating common and specific features:* We extract the common feature $C_{i}$ and specific feature $S_{i}$ of the compound from the common space and the specific space, respectively. Recognizing that both features contribute valuable information for predicting MoA, we construct a two-layer fully connected neural network as the fusion layer. The fused fingerprint feature $F_{i}^{fp}$ captures both common and specific compound structural characteristics, providing a comprehensive and compact representation.

### 2.3 MoA predictor and comprehensive loss function

**Figure 3.**
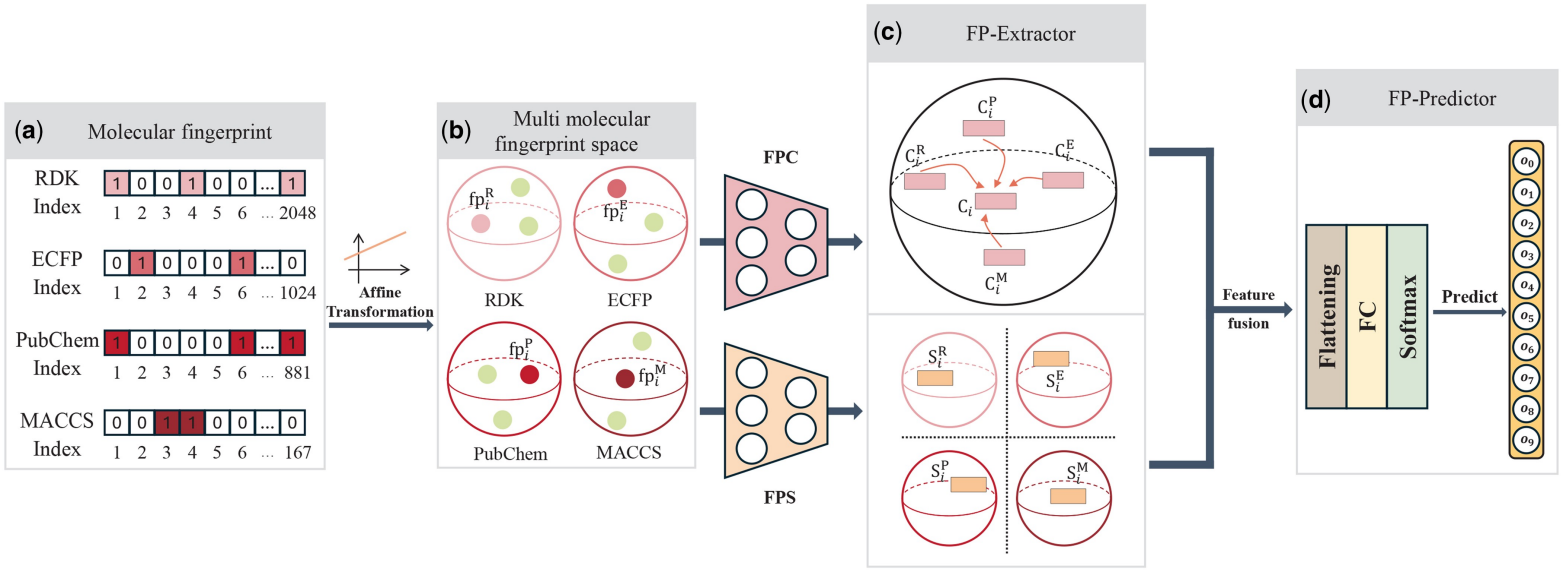
Another branch using fingerprint data to predict MoA, including (a) molecular fingerprint, four distinct types of molecular fingerprints, (b) multi molecular fingerprint space, after affine transformation, compounds are distributed in different semantic spaces, (c) FP-Extractor, a fingerprint feature extractor, and (d) FP-Predictor, an independent MoA classification predictor for extracted fingerprint features, where $o_{0},\ldots ,o_{9}$ represent the predicted probabilities for the 10 MoA categories.

During the training process, the feature extraction and prediction for each modality are performed independently. As shown in Figs 2c and 3d, the cell image predictor (CI-Predictor) and the fingerprint predictor (FP-Predictor) incorporate a linear layer with a softmax layer as a classifier, and they input features extracted from cell images and fingerprints respectively to derive predictive probability scores ${\text{score}}^{\text{CI}}$ and ${\text{score}}^{\text{FP}}$. The cross entropy loss between the true distribution of MoA and the predicted score is,


(11) $$\begin{array}{c} L^{\text{CI}}=-\sum_{s\in S}y^{\text{true}}\times \text{log}({\text{score}}^{\text{CI}}),\\ L^{\text{FP}}=-\sum_{s\in S}y^{\text{true}}\times \text{log}({\text{score}}^{\text{FP}}), \end{array}$$

where S represents the set of all training samples, and $y^{\text{true}}$ denotes the true MoA class of the corresponding sample. The loss for CI-Extractor and CI-Predictor is denoted as $L^{\text{CI}}$, while the total loss for molecular FP-Extractor and FP-Predictor is given by $L^{\text{FP}}+L_{c}+L_{s}$.

The final prediction score of the MoA classification is the weighted sum of multimodal prediction socres, defined as


(12) $$\text{score}=\beta \times {\text{score}}^{\text{CI}}+(1-\beta )\times {\text{score}}^{\text{FP}},$$

where β is used to adjust the contributions of prediction scores from the two modalities.

## 3 Results

### 3.1 Performance evaluation

To facilitate performance comparison with other methods, we adopted the validation method proposed by Tian *et al.* (2023). We divided all compounds into 10 subsets based on class proportions, where each subset consists of matching image data and fingerprint representations of the compounds. One subset is reserved for the final testing, while the remaining subsets are used for 5-fold cross-validation. In each validation iteration, eight subsets are used as the training set, and one subset is used as the validation set. We evaluate performance using accuracy, macro F1-score, and weighted F1-score, with detailed formulas provided in the Supplementary Section S2.

### 3.2 Implementation and parameter settings

Our proposed model is implemented with Python 3.10 and PyTorch 12.1, and it is trained on four NVIDIA GeForce RTX 3090 graphic cards with 24G graphic memory within an Ubuntu 20.04 environment. In the CI-Extractor and CI-Predictor modules, we set the number of epochs to 100, the learning rate to 0.001, and the dimension of final image feature to 512. In the FP-Extractor and FP-Predictor modules, the number of epochs is set to 100, the learning rate is set to 0.00001, the dimensions of the common space and specific space are both defined as 512, and the fused fingerprint feature dimension is 64. Furthermore, we invoke a callback function to reduce the learning rate if the learning progress of the model stalls due to excessively high learning rates, thereby enhancing model performance. For parameter selection, we first defined reasonable parameter ranges based on prior knowledge and empirical experience. Then, we used a grid search strategy to identify the optimal hyperparameters. The scope of hyper-parameters is provided in Supplementary Table S7.

### 3.3 Comparison with other methods

To comprehensively evaluate the predictive performance of the proposed method, we conducted a systematic comparison across three categories of state-of-the-art data-driven approaches: (i) image-based network methods, including GapNet (Hofmarcher *et al.* 2019), ShuffleNet (Zhang *et al.* 2018), DenseNet (Huang *et al.* 2017), ResNet (He *et al.* 2016), and EfficientNet (Tan and Le 2019); (ii) compound structure learning methods, such as GCN (Kipf and Welling 2016), GAT (Veličković *et al.* 2017), FPNN and FP-GNN (Cai *et al.* 2022), where FPNN utilizes encoded multi-modal molecular fingerprint features concatenated together as input to a fully connected neural network for training; and (iii) multimodal data processing methods, exemplified by Tian *et al.*’s method (Tian *et al.* 2023). Compared to other methods (Table 1), IFMoAP achieves the best performance with accuracy of 0.941, macro-F1 score of 0.938, and weighted-F1 score of 0.941. Tian *et al.*’s method achieves the second-best results with accuracy of 0.930, macro-F1 score of 0.920, and weighted-F1 score of 0.930. Both methods utilize two modalities of data, and the complementary information from perturbed cell images and compound structures demonstrates the significant potential of using multi-modal data in the context of MoA classification. Moreover, our proposed MRes-Net and FP-CS achieve state-of-the-art performance on single-modal data, with a detailed comparative analysis provided in Supplementary Section S3. Supplementary Table S8 presents a comparison between IFMoAP and other methods based on prediction values using the paired Wilcoxon test. The experimental results demonstrate that IFMoAP achieves statistically significant differences in prediction performance, highlighting its effectiveness and robustness.

**Table 1.** The comparison of IFMoAP with other three categories of methods in accuracy (Acc), macro-F1 score (M-F1), and weighted-F1 score (W-F1). Bold values indicate the best performance.

Data	Method	Acc	M-F1	W-F1
Multi modal	IFMoAP	**0.941**	**0.938**	**0.941**
	Tian *et al.*’s method	0.930	0.920	0.930
Cell image	MResNet	**0.824**	**0.821**	**0.825**
	EfficientNet	0.810	0.810	0.810
	ResNet	0.806	0.795	0.797
	DenseNet	0.793	0.781	0.787
	ShuffleNet	0.759	0.753	0.756
	GapNet	0.698	0.686	0.696
Compound structure	FP-CS	**0.733**	**0.699**	**0.715**
	FP-GNN	0.675	0.656	0.664
	FPNN	0.617	0.583	0.604
	GCN	0.558	0.481	0.500
	GAT	0.542	0.439	0.467

### 3.4 Ablation experiments on multimodal data

To investigate the importance of multimodal data for MoA prediction, we presented the results in Table 2, which demonstrate the predictive performances of both multimodal and unimodal data. Among all the tested samples, the model trained on multimodal data achieves the highest accuracy, macro-F1 score, and weighted-F1 score, with values of 0.941, 0.938, and 0.941, respectively. The result demonstrates that sensibly integrating multimodal data enhances the performances of prediction tasks. When using unimodal data as input, the cell image data outperformed the molecular fingerprint data on MoA prediction. The accuracy, macro-F1 score, and weighted-F1 score for cell image data are 0.824, 0.821, and 0.825, respectively, which are 9.1%, 12.2%, and 11% higher than those of the molecular fingerprint data. The result could be attributed to the targeted nature of the Cell Painting experiment, where the perturbed cellular morphology exhibits improved ability to express the functionality of the compounds compared to fingerprints. In addition, Supplementary Table S1 shows the accuracy of predictions for each MoA category using multimodal and unimodal data. The ablation experiments of CI-Extractor and FP-Extractor are presented in the Supplementary Section S4. A series of experiments is conducted to validate the effectiveness of the proposed modules.

**Table 2.** Ablation experiment of the multimodal data. Bold values indicate the best performance.

Cell image	Fingerprint	Acc	M-F1	W-F1
**✓**	×	0.824	0.821	0.825
×	√	0.733	0.699	0.715
√	√	**0.941**	**0.938**	**0.941**

### 3.5 Visual analysis of compound features

**Figure 4.**
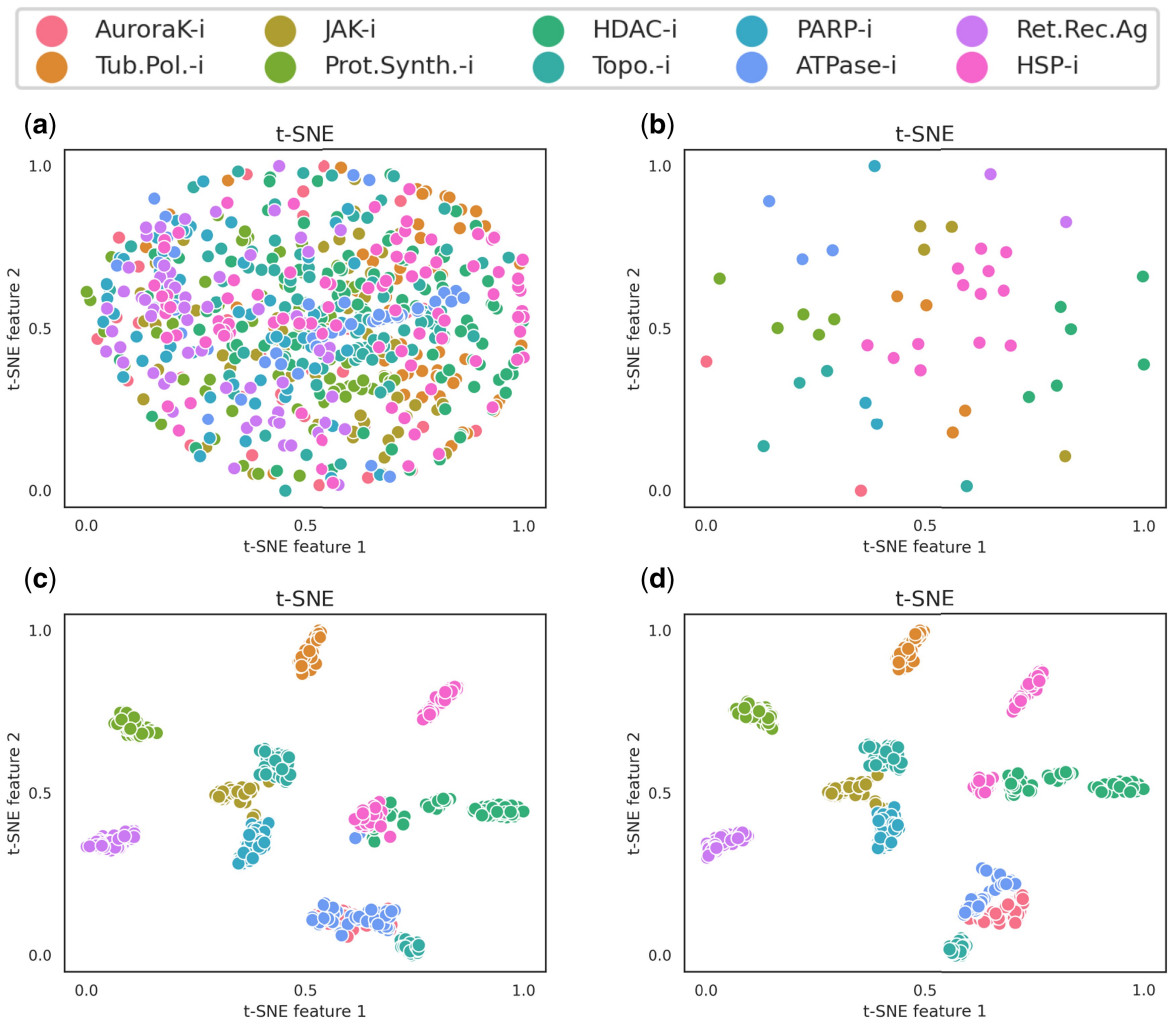
Distribution of t-SNE from compounds based on different feature representations, including (a) the raw perturbed cell image pixels, (b) the extracted fingerprint features, (c) the extracted cell image features, and (d) the multimodal features. Distinct colored scattered points represent various MoA classes of compounds.

As shown in Fig. 4, we visualized the low-dimensional features learned by the models using the t-SNE technique to validate their expressive powers. Figure 4a represents the visualization results of test samples’ features with perturbed cell image pixels as the input to the t-SNE algorithm. The scattered points representing compounds appear unordered. Figure 4b and c depict the visualization results of sample points with features extracted by FP-Extractor and CI-Extractor, respectively. Due to the one-to-many relationship between compounds and perturbed images, the sample points in Fig. 4b exhibit sparse overlapping, but it is still possible to observe the clustering of compounds from different MoA classes. In Fig. 4c, the classification of compounds is more evident, where the clusters of HSP-i and HDAC-i overlap, while ATPase-i and AuroraK-i are not separated. Figure 4d shows the visualization results after concatenating the image features and fingerprint features. The categories that are not well separated in Fig. 4c show improvement, further demonstrating the complementarity of the two modalities of data.

### 3.6 Exploration of multi-channel cell organelle image relationship

Compound perturbed cell images contain rich cellular morphology information, where each channel of the image records different organelles or cellular components. The mechanism of action of a compound can directly or indirectly cause changes in cellular morphology. Due to the diverse functions of different compounds, they may induce variations in different organelles or cellular components. The five channels in cell images correspond to the following organelles: (i) nucleus (DNA), (ii) mitochondria (Mito), (iii) F-actin cytoskeleton, golgi and plasma membrane (AGP), (iv) nucleoli and cytoplasmic RNA (RNA), and (v) the endoplasmic reticulum (ER). We aim to explore the relationship between them. We used MRes-Net to directly extract features from single-channel images and made predictions. The experimental results are shown in Table 3, and the model based on the Mito-channel images achieved the highest prediction values across seven classes (AuroraK-i, HDAC-i, HSP-i, PARP-i, Prot.Synth.-i, Ret.Rec.Ag, and Tub.Pol.-i) when considering solely individual channel images, and ER-channel and DNA-channel obtained the highest predictions in two different classes. The average prediction values of the aforementioned three channels also rank the top three, while the average prediction accuracy of the remaining two channels is below 0.6. The experimental findings suggest that mitochondria exhibit a higher sensitivity in expressing the MoA of compounds in comparison to other cellular organelles.

**Table 3.** Accuracy of each MoA class on model trained on single-channel cell images. Bold values indicate the best performance.

Organelle	All	DNA	Mito	AGP	RNA	ER
*MoA*						
ATPase-i	0.473	0.450	0.567	0.557	0.540	**0.570**
AuroraK-i	0.693	0.260	**0.860**	0.107	0.143	0.350
HDAC-i	**0.933**	0.692	0.887	0.753	0.743	0.765
HSP-i	**0.749**	0.607	0.700	0.653	0.667	0.664
JAK-i	**0.860**	0.503	0.107	0.083	0.200	0.140
PARP-i	**0.963**	0.457	0.947	0.840	0.557	0.703
Prot.Synth.-i	**0.997**	0.790	0.790	0.503	0.520	0.580
Ret.Rec.Ag	0.993	0.633	**0.997**	0.543	0.417	0.973
Topo.-i	0.638	0.724	0.653	0.589	0.742	**0.756**
Tub.Pol.-i	0.967	0.837	**0.983**	0.963	0.970	0.973

The observation aligns with the trend observed in studies focused on compound properties, which frequently utilize mitochondria-related data as a foundational basis for research (Zhang *et al.* 2017, Yu *et al.* 2024). Mitochondria are the center of cellular energy metabolism, and their function is susceptible to the effects of drug properties including MoA, leading to cell energy imbalances and potentially severe outcomes such as metabolic disruptions or increased toxicity (Morén *et al.* 2016, Zolkipli-Cunningham and Falk 2017). However, the prediction results for the JAK inhibitors by the Mito-channel are very low, with an accuracy of only 0.107. The possible reason is that the diverse changes in diverse cellular changes induced by the JAK inhibitors make it challenging for the model to learn consistent features solely based on mitochondria. Image models trained on five channel images achieved a prediction accuracy of 0.860 for the JAK inhibitors. This may be due to the interference of redundant information from multiple channels, which hinders the model’s ability to focus on the specific organelles or cellular components that are more relevant to a particular MoA. However, the prediction results for certain MoAs suggest that multi-channel images may not perform as well as single-channel. Consequently, experiments can be conducted to extract images of relevant organelles for specific tasks to optimize cost-effectiveness.

### 3.7 Exploration in the realm of fingerprint space

Compound structure represents a distinct data modality from perturbed cell images, with various fingerprint definitions. We visualized the features extracted from two perspectives that can represent compounds. Supplementary Figure S1a shows that compounds represented by common features and specific features are clearly separated in the 2D space. The visualization result confirms the substantial impact of our design on fingerprint feature extraction. Additionally, we examined four types of fingerprint embeddings in both the common space and specific space. In Supplementary Fig. S1b, the four types of fingerprint embeddings in the common space do not show clear distinctions, whereas in Supplementary Fig. S1c, the compound scatter plots in the specific space distinctly separate into four clusters. The fingerprint features in the common space exhibit consistency, while the specific features are relatively independent. A detailed discussion of two types of fingerprint comparative experiments is provided in the Supplementary Section S6.

### 3.8 Exploration of MoA predictions and compound correlations

We conducted an analysis encompassing ten MoA categories and 24 compounds from the test set to further elucidate the predictive capacity of IFMoAP for MoA prediction of compound. The confusion matrix based on the IFMoAP prediction results, as illustrated in Supplementary Fig. S2a, reveals that the true positive rates for nine MoAs exceed 90%, with JAK-i exhibiting a marginally lower rate. In Supplementary Table S4, specific prediction results for 24 compound categories are provided. Due to the availability of multiple samples for a single compound, the final classification result for that compound is determined by a voting method. The results indicate that 23 copounds were predicted accurately, with one compound being incorrectly predicted. Further details can be found in Supplementary Section S7.

### 3.9 Case study

We conducted a case analysis on compound CBK290717 (curcumol), which exhibits the lowest prediction accuracy, to explore the reasons for misclassification. Supplementary Table S4 indicates that the compound was originally classified as a Janus Kinase inhibitor but was predicted as PARP-i and Topo.-i. Confusion matrices calculated based on cell image and fingerprint data-driven model predictions are presented in Supplementary Fig. S2b and c. Supplementary Figure S2b illustrates instances where JAK-i was misclassified as PARP-i, while Supplementary Fig. S2c shows cases where JAK-i was wrongly predicted as Topo-i. The average of the 30 × 30 matrix values in Supplementary Fig. S4a is extracted as the similarity correlation value between two compounds, and the new heatmap matrix in Supplementary Fig. S4b is obtained by filling the values. From the figure, we identified four compounds with the highest similarity to curcumol, namely CBK289987 (NU-1025), CBK308120 (EB-47), CBK308819 (TAS-103), and CBK290529 (amonafide). The first two compounds are PARP-i inhibitors, while the latter two are Topo-i inhibitors. These two types of inhibitors function by disrupting DNA repair and replication processes, leading to the accumulation of DNA damage and subsequent induction of cell death. Several studies have indicated that curcumol may exert certain effects on cellular DNA, including the induction of DNA damage or interference with DNA repair processes, particularly in cancer cells (Cai *et al.* 2017, Peng *et al.* 2018, Hashem *et al.* 2021). Consequently, cell perturbation effects similar to those of PARP and topoisomerase inhibitors may be the main reason for curcumol’s inability to classify correctly based on cell images.

## 4 Conclusion and perspectives

We integrated multimodal data and introduced the IFMoAP model for predicting the MoA of compounds. Two extractors were proposed to extract cell image features and fingerprint features of compounds. The modified ResNet not only effectively extracted features from five-channel cell images but also integrated cross-scale image features. The fingerprint feature extractor was designed with the aim of exploring the commonalities and specificities among four types of fingerprints. Targeted modules and loss functions were devised to capture the common and specific features of fingerprints, which were then fused together. The synergy of knowledge from two distinct domains has enhanced the predictive performance of MoA of the compound. Comparative experiments with three category models demonstrated IFMoAP’s superior performance, with further confirmation of the effectiveness of the proposed modules through ablation studies. Explorations and visualizations showcased the efficacy of the extracted features by revealing the underlying biological mechanisms. The case analysis revealed that prediction errors may be attributed to the existing modalities being insufficient to differentiate similar compounds. Overall, a holistic approach that embraces interdisciplinary data integration and cutting-edge methodologies will be pivotal in advancing the predictive capabilities in drug design and mechanism elucidation.

## Supplementary Material

btaf223_Supplementary_Data
