# Supplementary material for: Synergizing multimodal data and fingerprint space exploration for mechanism of action prediction
Source: Bioinformatics. 2025 Jun 3;41(6):btaf223. doi: 10.1093/bioinformatics/btaf223 (PMC12145173; doi:10.1093/bioinformatics/btaf223)
Supplement: btaf223_Supplementary_Data [file btaf223_supplementary_data.pdf]

Supplementary file for "Synergizing multimodal data and fingerprint space exploration for mechanism of action prediction"

Kaimiao Hu, Jianguo Wei, Changming Sun, Jie Geng, Leyi Wei*, Qi Dai*, and Ran Su*

1.Dataset details and data preprocessing 2

2. Performance evaluation 3

3. Comparison with other methods 4

4. Ablation experiments 5

6. Exploration in the realm of fingerprint space 7

7. Exploration of MoA predictions and compound correlations 8

8. Supplementary Tables 10

9. Supplementary Figures 12

1. Dataset details and data preprocessing

Tian et al. applied the Cell Painting technique by treating compounds at a dose of 10 micromoles in a 384-well plate format with U2OS cells. They employed the ImageXpress Micro XLS automated microscope to capture cell images from the multi-well plates, acquiring five-channel cell images. These images contain morphological information of cellular structures perturbed by compounds, including the nucleus, nucleolus, cytoplasmic RNA, endoplasmic reticulum, Golgi apparatus, plasma membrane, and actin cytoskeleton.

We apply the RDKit library to parse and process compounds represented in the SMILES format, converting them into four types of molecular fingerprints:

i. RDK fingerprint (2048-bit) based on chemical bonds and substructures, used for describing the structure and features of molecules;

ii. ECFP fingerprint (1024-bit) based on molecular topology to capture intermolecular connectivity;

iii. PubChem fingerprint (881-bit) derived from the PubChem database to describe the structures and properties of compounds;

iv. MACCS fingerprint (167-bit) primarily employed to delineate molecular substructures and fundamental features.

The compounds involved in this study are classified into one of ten MoA classes, which are defined as follows:

1. ATPase Inhibitor: Compounds that inhibit ATPase activity, disrupting cellular energy metabolism.
2. Aurora Kinase Inhibitor: Compounds targeting aurora kinases, which are key regulators of mitosis, leading to cell division arrest.
3. HDAC Inhibitor: Compounds that inhibit histone deacetylases, affecting gene expression and promoting cell cycle arrest or apoptosis.
4. HSP Inhibitor: Compounds targeting heat shock proteins, which are crucial for protein folding and cellular stress responses.
5. JAK Inhibitor: Compounds inhibiting Janus kinases, which play a vital role in cytokine signaling pathways.
6. PARP Inhibitor: Compounds targeting poly (ADP-ribose) polymerase, impairing DNA repair mechanisms and inducing cell death in cancer cells.
7. Protein Synthesis Inhibitor: Compounds that block ribosomal function, halting protein synthesis.
8. Retinoid Receptor Agonist: Compounds that activate retinoid receptors, influencing cell differentiation and proliferation.

9. Topoisomerase Inhibitor: Compounds that interfere with DNA topoisomerases, leading to DNA damage and cell death.
10. Tubulin Polymerization Inhibitor: Compounds disrupting tubulin dynamics, impairing microtubule assembly necessary for cell division.

The five channel image data of Cell Painting adopts a standardization method to eliminate the plate-level effect. This method is based on the control group cell image (DMSO Well). The method calculates the average and standard deviation of pixels for each channel to standardize the data and adjusts the image from the original 2160×2160 pixels to 256×256 pixels. The complete image dataset contains 7,710 five-channel cellular images. After excluding control group images reserved, 6,990 images remain available for experiments.

2. Performance evaluation

For a specific class k, TP^k, FP^k, TN^k and FN^k are defined as follows:

- TP^k represents the samples correctly predicted as k-th class out of the actual k-th class samples.
- FP^k represents the samples inaccurately predicted as k-th class out of samples not belonging to k-th class.
- TN^k represents the samples accurately predicted as not k-th class out of samples not belonging to k-th class.
- FN^k represents the samples inaccurately predicted as not k-th class out of the actual k-th class samples.

Based on these terms, we can calculate precision (P) and recall (R) of k-th class as follows:

$$\mathrm{P}^k = \frac{\mathrm{TP}^k}{\mathrm{TP}^k+\mathrm{FP}^k},\ \mathrm{R}^k = \frac{\mathrm{TP}^k}{\mathrm{TP}^k+\mathrm{FN}^k}.$$

From these calculations, we obtain the F1 score of k-th class,

$$\mathrm{F1}^k = \frac{2\times\mathrm{P}^k\times\mathrm{R}^k}{\mathrm{P}^k+\mathrm{R}^k}.$$

Finally, we utilize the F1 score across all classes to compute both the macro-F1 score and the weighted-F1 score,

$$\text{macro F1} - \text{score} = \frac{1}{n}\sum_{k=1}^{n}\mathrm{F1}^k,$$

$$\text{weighted F1} - \text{score} = \sum_{k=1}^{n}\mathrm{w}^k\mathrm{F1}^k,$$

where n is the total number of classes, and w^k is the proportion of the k-th category in the total sample. In addition, we also considered the micro F1-score, which is derived from the global precision and recall calculations:

$$P_{micro} = \frac{\sum_{k=1}^{n} \mathrm{TP}^k}{\sum_{k=1}^{n}(\mathrm{TP}^k+\mathrm{FP}^k)},$$

$$R_{micro} = \frac{\sum_{k=1}^{n} \mathrm{TP}^k}{\sum_{k=1}^{n}(\mathrm{TP}^k+\mathrm{FN}^k)},$$

$$\text{micro F1} - \text{score} = \frac{2\times P_{micro}\times R_{micro}}{P_{micro}+R_{micro}}.$$

Since all predicted samples are either correctly classified or misclassified, we can derive the following equalities:

$$\sum_{k=1}^{n}(\mathrm{TP}^k + \mathrm{FP}^k) = N,$$

$$\sum_{k=1}^{n}(\mathrm{TP}^k + \mathrm{FN}^k) = N.$$

where N is the total number of samples. In the multi-class classification tasks, accuracy is generally defined as the ratio of correctly predicted samples to the total number of samples, expressed as,

$$\text{Accuray} = \frac{\text{Number of correct classifications}}{\text{Total number of samples}} = \frac{\sum_{k=1}^{n} \mathrm{TP}^k}{N}.$$

which is mathematically equivalent to the micro F1-score in a multi-class classification setting. Therefore, in our study, we have chosen to report accuracy along with macro F1-score and weighted F1-score, as micro F1-score does not provide additional information beyond accuracy.

3. Comparison with other methods

In comparison to several classical network methods, MRes-Net achieves an accuracy of 0.824, macro-F1 score of 0.821, and weighted-F1 score of 0.825 (Table 1). EfficientNet, ResNet, and DenseNet also demonstrate good prediction accuracies with values of 0.810, 0.806, and 0.793, respectively. However, Shuffle-Net performs relatively poorer in terms of prediction accuracy. These results indicate that the modified modules in MResNet are more suitable for extracting cell features, leading to improved prediction outcomes. On the other hand, GAP-Net, which is used for predicting compound activity, only achieves an accuracy of 0.669, macro-F1 score of 0.661, and weighted-F1 score of 0.672. This may be attributed to the network structure being more suitable for binary classification problems and its lack of pre-trained parameters, resulting in poor transferability.

Our proposed FP-CS method achieves an accuracy of 0.733, macro-F1 score of 0.699, and weighted-F1 score of 0.715, which is an improvement over FPNN. This

indicates that the two complementary features extracted by the designed encoders make significant contributions to MoA prediction. The GCN model and the GAT model that consider the compound graph structures achieve accuracies of 0.558 and 0.525, respectively, while the FP-GNN model, which combines fingerprint and graph structure information, improves the accuracy to 0.630, but still falls short compared to FP-CS. This suggests that both fingerprint and graph features can represent compounds, and their fusion also benefits performance improvement, but they are still not as representative as the complementary features captured by multiple fingerprints.

4. Ablation experiments

Ablation experiments on CI-Extractor. In ResNet-50, we captured multi-scale cell morphological features by modifying STAGE 0, cascaded coarse features and fine features, and input them into an attention mechanism at the granularity-level. The final cell image features were then fed into a fully connected classifier to predict scores. To systematically evaluate the impact of these modules, we conducted ablation studies as detailed in Supplementary Table S2. Changing only the number of input channels in ResNet-50 achieved an accuracy of 0.806, a macro-F1 score of 0.795, and a weighted-F1 score of 0.797.

(i) Modified STAGE 0. Our customized modification to STAGE 0 enhances the model's capability in capturing features, resulting in notable improvements of 1.1% in accuracy, 1.7% in macro-F1 score, and 1.9% in weighted-F1 score. This underscores the benefit of enlarging the receptive field and incorporating multi-scale information for effective feature extraction.

(ii) Image feature concatenation. The concatenation of cascade coarse and fine features further boosts accuracy by 1.0%, indicating the significance of shallow features in representing cellular morphology. This integration provides a more intuitive reflection of the characteristics pertinent to the MoA classes, thereby enriching the feature representation.

(i) + (ii). The predictive performance of the model integrating enhancements described above is further enhanced, surpassing the accuracy of using a single module by 0.5$\%$ using the approach in (i) and 0.6% using the approach in (ii) respectively. Furthermore, a substantial enhancement is observed in both macro-F1 and weighted-F1 scores.

(i) + (ii) + attention. Our final CI-Extractor, combined all moudules, achieves superior performance across all evaluation metrics when only feding image data for model

inference. Specifically, it surpasses the baseline by substantial margins (accuracy: from 0.806 to 0.812, macro-F1 score: from 0.795 to 0.810, weighted-F1 score: from 0.797 to 0.811), demonstrating the efficacy of the proposed architectural modifications and feature fusion strategy.

Ablation experiments on FP-Extractor. We explored the commonalities and specificities of compounds from different spaces and extracted common features and specific features from multiple types of fingerprints. We then fused these fingerprint features and performed predictions. Supplementary Table S3 shows the ablation experimental results of the components in FP-Extractor.

(i) Only common feature. When considering only the features extracted by the FPC encoder for MoA prediction, we obtained modest results, with accuracy, macro-F1 score, and weighted-F1 score of 0.667, 0.622, and 0.650, respectively.

(ii) Only specific feature. The specific features achieves better results compared to the common features, with the accuracy, macro-F1 score, and weighted-F1 score being 0.708, 0.669, and 0.691, respectively. This may be attributed to the richer variety of fingerprint information contained in the specific space compared to the common space.

(i) + (ii) + feature concatenation. We directly predicted MoA of compounds by concatenating the two types of features. The results indicated no improvement on accuracy, but there were a slight improvements on the other two metrics.

(i) + (ii) + feature fusion: The complete fingerprint prediction model with a fusion layer achieves the best performance in this branch, with an accuracy of 0.733.

These findings underscore the importance of considering both common and specific features when studying compound characteristics and predicting MoA. The specific space captures a more diverse range of fingerprint information, leading to improved performance compared to the common space alone. While the concatenation of common and specific features did not significantly enhance accuracy in this case, it resulted in a slight improvement in the other two metrics. The fingerprint feature integration method utilizing the fusion layer demonstrated the best performance, highlighting the effectiveness of a fusion approach in fingerprint prediction. These results have implications for drug discovery and development, emphasizing the significance of exploring fingerprint information from multiple perspectives to enhance the understanding of compound properties and improve prediction accuracy.

6. Exploration in the realm of fingerprint space

To evaluate the contributions of different types of fingerprints to MoA prediction, we devised two types of fingerprint comparative experiments: single-type fingerprint experiments and single-type fingerprint ablation experiments. The former involves inputting only one type of fingerprint into a model based on a fully connected encoder for prediction, while the latter entails systematically excluding one type of fingerprint input into the FP-CS module each time. Both of these experimental approaches aid in assessing the impact of each fingerprint on the experimental outcomes, as shown in Supplementary Figure S1(d), Supplementary Table S5, and Supplementary Table S6.

In the single-type fingerprint experiments, the results indicated that PubChem fingerprints achieved the best prediction performance, with accuracy, macro-F1 score, and weighted-F1 score being 0.625, 0.593, and 0.661, respectively. The ECFP and MACCS fingerprints exhibited comparable efficacy, whereas the model's performance was notably diminished when utilizing the RDK fingerprints, attaining a modest accuracy of 0.550. This performance disparity suggests that PubChem fingerprints encapsulate richer and more comprehensive semantic information, making them more effective in capturing key molecular characteristics. In contrast, RDK fingerprints, despite their structural representation advantages, may not sufficiently capture the chemical and biological properties critical for MoA classification. The well-defined and semantically rich data representation enhances feature extraction and model interpretation, leading to improved predictive performance.

A closer examination of MoA-specific classification performance reveals that the suitability of different fingerprints varies significantly across MoA classes. For example, ATPase inhibitors are best classified using PubChem and MACCS fingerprints, likely due to their ability to capture bioactivity-related substructures that are characteristic of this category. Aurora kinase inhibitors, in contrast, exhibit significantly better performance with ECFP fingerprints, suggesting that the circular substructure-based representation is well-suited for identifying kinase-inhibitor interactions. Interestingly, HDAC inhibitors demonstrate high classification accuracy with both RDK and ECFP fingerprints, indicating that these fingerprints effectively capture the key chemical scaffolds associated with histone deacetylase inhibition. Conversely, MoA classes such as JAK inhibitors, PARP inhibitors and Tubulin Polymerization inhibitors exhibit poor classification performance across all single-fingerprint models, with accuracy values generally below 0.5. This suggests that these classes may require more complex molecular representations or additional biological context, which a single fingerprint type alone may not adequately provide. These

observations reinforce the necessity of integrating multiple fingerprint types to enhance model robustness and generalizability.

In the single-type fingerprint ablation experiments, the model demonstrating the highest predictive performance (accuracy of 0.725) was obtained by ablating the RDK fingerprint. This finding is consistent with the weak predictive capability of RDK fingerprints in the single-type fingerprint experiments, suggesting that RDK contributes the least distinctive information among the four types. The models of ablation of ECFP, PubChem, and MACCS achieved promising results with the accuracies of 0.650, 0.683 and 0.683, respectively. Further analysis of MoA-specific ablation effects (Supplementary Table 2) reveals that certain MoA classes are more sensitive to specific fingerprint removals. For instance, the removal of ECFP fingerprints leads to a substantial drop in the classification accuracy of Aurora kinase inhibitors, reinforcing its critical role in capturing kinase-related features. Similarly, ablating PubChem fingerprints reduces accuracy for ATPase inhibitors, confirming its contribution to bioactivity recognition. These findings further support the hypothesis that different fingerprint types encode distinct and complementary molecular information.

The performance in single-type fingerprint ablation experiments surpassed that of single-type fingerprint experiments, affirming that not only can multimodal data provide complementary information, but diverse types of fingerprints within a single modality can also enrich the representation of compounds.

7. Exploration of MoA predictions and compound correlations

We conducted an analysis encompassing ten MoA categories and 24 compounds from the test set to further elucidate the predictive capacity of IFMoAP for MoA prediction of compound. The confusion matrix based on the IFMoAP prediction results, as illustrated in Supplementary Figure S2(a), reveals that the true positive rates for nine MoAs exceed 90%, with JAK-i exhibiting a marginally lower rate. In Supplementary Table S4, specific prediction results for 24 compound categories are provided. Due to the availability of multiple samples for a single compound, the final classification result for that compound is determined by a voting method. The results indicate that 23 compounds were predicted accurately, with one compound being incorrectly predicted. Supplementary Figure S3 illustrates the prediction hit scores across all compound samples. Notably, 19 compounds achieved a perfect probability score of 1 for their top prediction (top 1). Additionally, two compounds attained a cumulative probability of 1 within their top 2 predictions, while other two compounds reached this perfect score within their top 3 predictions.

To delve further into the interrelations among compounds, the similarity heatmap matrix was computed based on the extracted cellular image features of compounds. As illustrated in Supplementary Figure S4(a), the x-axis represents the image feature representations of compounds, with each small block of 30 units indicating the image feature representations of a specific compound. Enhanced prominence of the small blocks along the diagonal in the matrix signifies a robust correlation in the perturbation of cellular images induced by a particular compound across distinct plates and wells. The similarity heatmap provides a more intuitive visualization of the morphological changes in cells following compound perturbation, indirectly indicating their functionality. For instance, the three compounds exhibiting the most similar image features to CBK277957 (HDAC-i) after cellular perturbation are CBK289740, CBK277961, and CBK290547. Notably, these are also HDAC inhibitors, demonstrating that the morphological characteristics induced by these inhibitors are distinctive. However, there are cases where compounds with similar perturbation effects do not share the same MoA.

8. Supplementary Tables

Supplementary Table S1. Accuracy of each MoA class on different models.

MoA \ Model	IFMoAP	MResNet	FP-CS
ATPase-i	0.947	0.473	1.000
AuroraK-i	0.997	0.693	0.900
HDAC-i	1.000	0.933	1.000
HSP-i	0.889	0.749	0.467
JAK-i	0.750	0.860	0.500
PARP-i	0.990	0.963	0.500
Prot.Synth.-i	1.000	0.997	1.000
Ret.Rec.Ag	1.000	0.993	1.000
Topo.-i	0.864	0.638	0.667
Tub.Pol.-i	0.980	0.967	0.200

Supplementary Table S2. Ablation experiment of the CI-Extractor.

STAGE0 (M)	Concat	Attention	Acc	M-F1	W-F1
×	×	×	0.806	0.795	0.797
√	×	×	0.817	0.812	0.816
×	√	×	0.816	0.806	0.810
√	√	×	0.822	0.819	0.822
√	√	√	**0.824**	**0.821**	**0.825**

Supplementary Table S3. Ablation experiment of the FP-Extractor.

FPC	FPS	Concat	Fusion	Acc	M-F1	W-F1
√	×	×	×	0.667	0.622	0.650
×	√	×	×	0.708	0.669	0.691
√	√	√	×	0.708	0.676	0.697
√	√	×	√	**0.733**	**0.699**	**0.715**

Supplementary Table S4. Prediction results of MoA categories for 24 compounds

	AuroraK-i	Tub.Pol.-i	JAK-i	Prot.Synth.-i	HDAC-i	Topo.-i	PARP-i	ATPase-i	Ret.Rec.Ag	HSP-i
CBK200949	3	0	0	0	0	27	0	0	0	0
CBK277957	0	0	0	0	30	0	0	0	0	0
CBK277961	0	0	0	0	30	0	0	0	0	0
CBK278064	30	0	0	0	0	0	0	0	0	0
CBK278556G	0	0	3	0	4	0	1	0	0	22
CBK288281	30	0	0	0	0	0	0	0	0	0
CBK289740	0	0	0	0	30	0	0	0	0	0
CBK289882	0	0	0	0	0	0	0	0	30	0
CBK289987	0	0	0	0	0	0	30	0	0	0
CBK290529	0	0	0	0	0	28	2	0	0	0
CBK290547	0	0	0	0	30	0	0	0	0	0
CBK290717	0	0	7	0	0	10	13	0	0	0
CBK290799	0	0	0	0	0	0	0	0	0	30
CBK290852	0	0	30	0	0	0	0	0	0	0
CBK291084	0	0	0	0	0	0	0	30	0	0
CBK303888	0	0	0	0	0	0	0	29	0	1
CBK307956	0	0	0	30	0	0	0	0	0	0
CBK308108	0	0	0	0	0	0	30	0	0	0
CBK308608	0	0	0	30	0	0	0	0	0	0
CBK308819	0	0	0	0	0	30	0	0	0	0
CBK308876	0	0	0	0	0	0	0	0	30	0
CBK309016	0	30	0	0	0	0	0	0	0	0
CBK309437	0	30	0	0	0	0	0	0	0	0
CBK309488	0	0	0	0	0	0	0	0	0	30

Supplementary Table S5. Accuracy of each MoA class on single-type fingerprint experiments

MoA \ FP	RDK	ECFP	PubChem	MACCS
ATPase-i	0.500	0.500	1.000	1.000
AuroraK-i	0.100	0.700	0.600	0.300
HDAC-i	1.000	1.000	0.800	0.467
HSP-i	0.000	0.600	0.333	0.500
JAK-i	0.500	0.000	0.500	0.500
PARP-i	0.500	0.500	0.500	0.500
Prot.Synth.-i	1.000	0.400	1.000	0.500
Ret.Rec.Ag	1.000	0.800	1.000	0.700
Topo.-i	0.600	0.733	0.533	0.600
Tub.Pol.-i	0.100	0.400	0.000	0.200
Accuracy	0.550	0.608	0.625	0.608

Supplementary Table S6. Accuracy of each MoA class on single-type fingerprint ablation experiments

MoA \ FP	RDK	ECFP	PubChem	MACCS
ATPase-i	1.000	0.900	0.500	0.900
AuroraK-i	1.000	0.300	0.600	0.700
HDAC-i	1.000	1.000	1.000	1.000
HSP-i	0.400	0.333	0.467	0.400
JAK-i	0.400	0.500	0.500	0.400
PARP-i	0.500	0.500	0.500	0.600
Prot.Synth.-i	0.900	1.000	1.000	1.000
Ret.Rec.Ag	1.000	1.000	1.000	1.000
Topo.-i	0.667	0.667	0.667	0.667
Tub.Pol.-i	0.300	0.100	0.400	0.000
Accuracy	0.725	0.650	0.683	0.683

Supplementary Table S7. The scope of the hyper-parameter values in IFMoAP.

Hyper-parameter	The scope of the hyper-parameter values
Epoch	[50, 75, 100, 125, 150]
Batch size	[16, 32, 64]
Learning rate in CI-Extractor	[0.001, 0.0001, 0.00001]
Learning rate in -Extractor	[0.001, 0.0001, 0.00001]
The dimension of final image feature	[256, 512,1024]

The dimension of fused common feature and specific feature	[256, 512,1024]
The dimension of fused fingerprint feature	[64,128,256]
β	[0.1-0.9]

Supplementary Table S8. Comparison between IFMoAP and other methods based on prediction values with the paired Wilcoxon test

MoA \ Method	Tian et al.'s method	MResNet	EfficientNet	ResNet	DenseNet	ShuffleNet	GapNet
ATPase-i	2.48e-24	1.05e-27	1.21e-04	3.10e-19	2.15e-11	1.97e-01	6.21e-03
AuroraK-i	1.44e-05	2.37e-27	2.89e-04	1.40e-15	1.18e-07	1.87e-01	6.98e-03
HDAC-i	4.23e-02	1.75e-01	3.42e-07	7.19e-40	6.91e-02	1.01e-04	5.69e-05
HSP-i	2.50e-09	5.96e-02	6.85e-12	5.00e-39	1.46e-01	1.92e-09	7.42e-02
JAK-i	3.29e-01	1.31e-01	9.70e-02	2.59e-19	8.03e-06	3.46e-02	1.55e-01
PARP-i	1.23e-10	1.69e-22	1.80e-01	3.36e-33	1.45e-01	2.06e-03	2.35e-07
Prot.Synth.-i	1.20e-01	6.58e-03	8.41e-04	3.27e-10	1.51e-01	6.31e-02	3.22e-03
Ret.Rec.Ag	9.66e-25	9.60e-12	9.87e-02	1.16e-21	9.91e-06	2.36e-02	1.83e-01
Topo.-i	9.94e-03	8.75e-02	1.14e-01	3.97e-35	9.36e-04	8.98e-03	8.17e-06
Tub.Pol.-i	3.02e-03	2.17e-08	3.80e-06	9.74e-28	2.10e-01	1.46e-01	1.21e-05

MoA \ Method	FP-CS	FP-GNN	FPNN	GCN	GAT
ATPase-i	1.05e-27	2.11e-01	3.07e-22	1.02e-15	1.38e-48
AuroraK-i	2.37e-27	1.91e-01	7.56e-04	1.15e-22	1.60e-13
HDAC-i	1.75e-01	7.00e-34	1.11e-01	8.60e-91	2.39e-05
HSP-i	5.96e-02	1.44e-01	3.71e-07	7.56e-47	5.00e-14
JAK-i	1.31e-01	2.34e-26	7.08e-02	1.50e-103	4.83e-02
PARP-i	1.69e-22	2.24e-14	3.99e-03	5.92e-43	1.69e-41
Prot.Synth.-i	6.58e-03	5.61e-03	1.64e-01	2.00e-63	6.51e-18
Ret.Rec.Ag	9.60e-12	3.67e-16	1.25e-01	3.16e-84	1.92e-51
Topo.-i	8.75e-02	7.16e-09	8.86e-02	1.21e-87	5.70e-08
Tub.Pol.-i	2.17e-08	5.84e-03	4.78e-02	6.37e-67	4.95e-39

9. Supplementary Figures

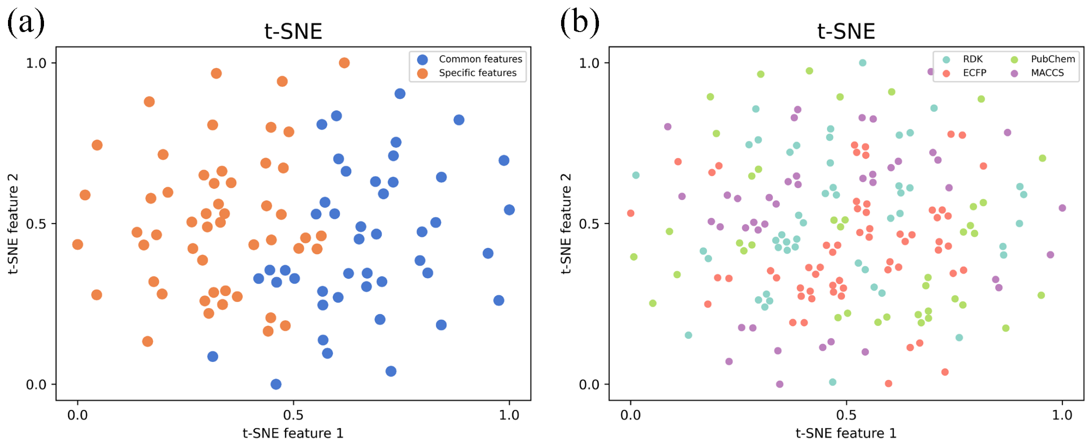

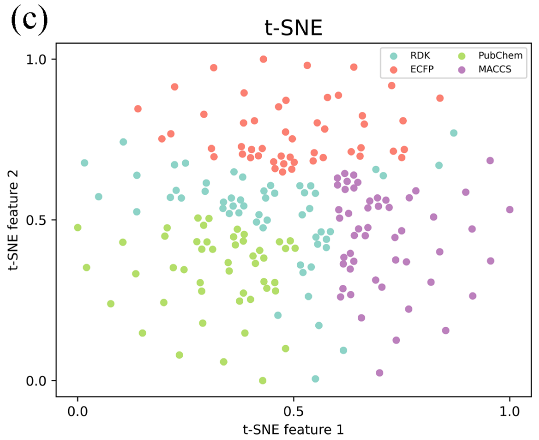

(d)

	RDK	ECFP	PubChem	MACCS
ACC	0.725	0.650	0.683	0.683
M-F1	0.698	0.611	0.668	0.632
W-F1	0.714	0.639	0.689	0.655
ACC	0.550	0.608	0.625	0.608
M-F1	0.501	0.553	0.593	0.569
W-F1	0.530	0.592	0.661	0.594

Supplementary Figure S1. Distribution of t-SNE of compound fingerprint features and experiments on fingerprint categories, including (a) the visualization of common features and specific features of fingerprint, (b) the visualization of four types of fingerprint features in a common space, (c) the visualization of four types of fingerprint features in a specific space, and (d) the single-type fingerprint ablation experiments and single-type fingerprint experiments (Blue background for the former experiment results, orange background for the latter experiment results).

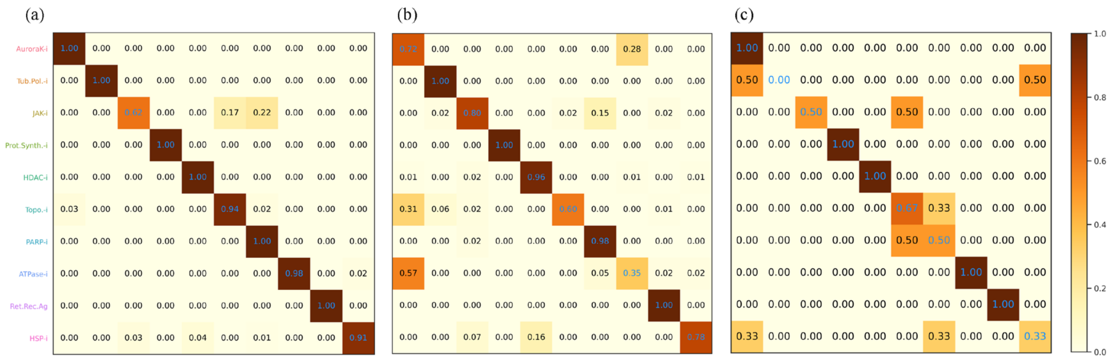

Supplementary Figure S2. Confusion matrix calculated based on predictions from models driven by different data sources, including (a) the multimodal data, (b) the cell perturbation images, and (c) the molecular fingerprints. The labels for x-axis and y-axis

of these matrices are both MoA categories.

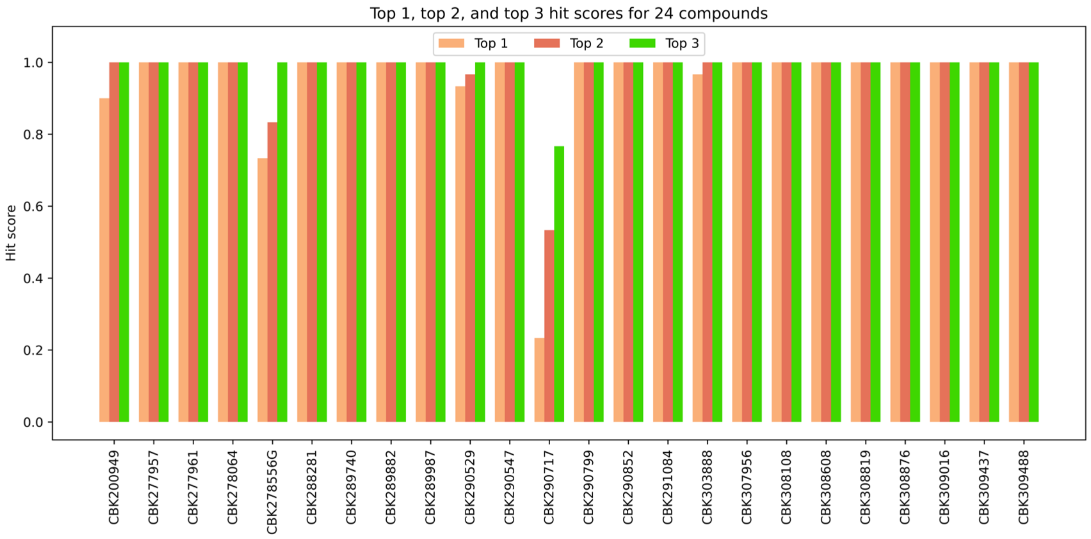

Supplementary Figure S3. Top-k hit scores for predicting MoA candidates of 24 compounds.

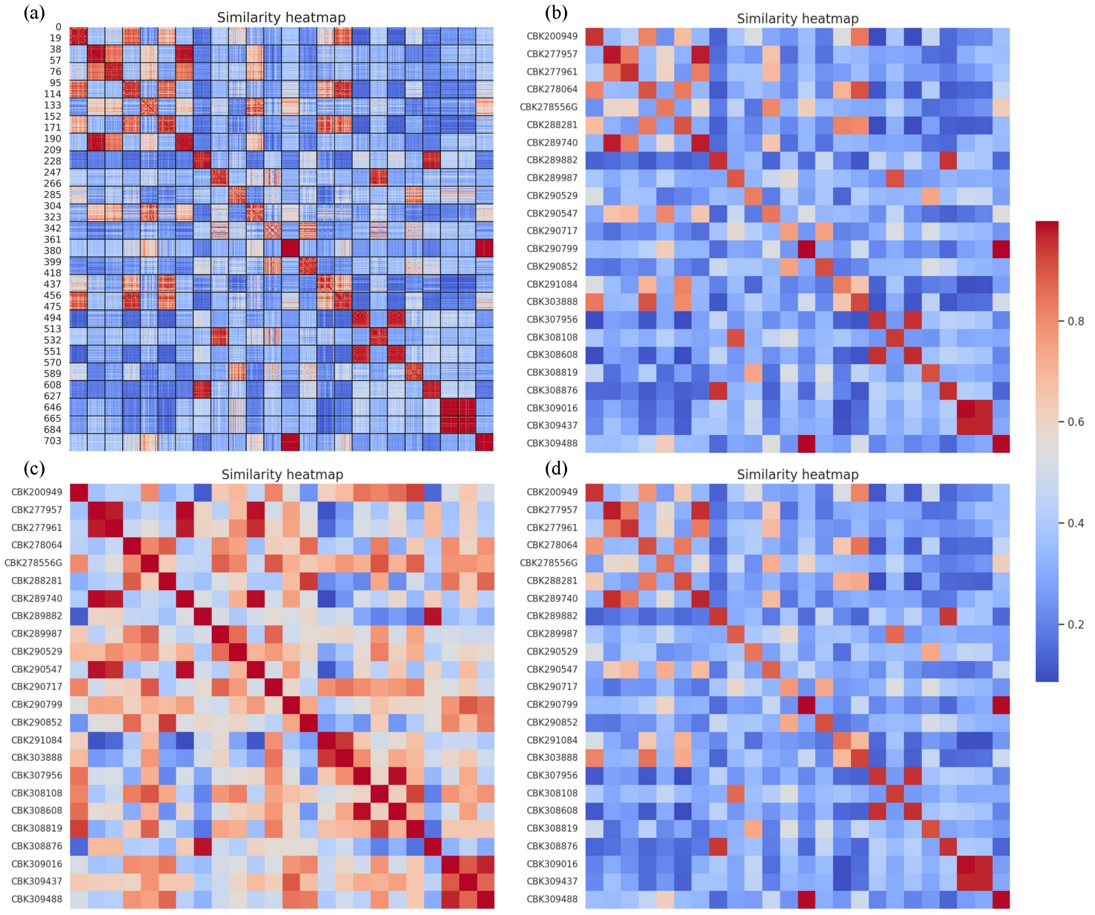

Supplementary Figure S4. Four similarity heatmaps, including: (a) computed based on extracted compound image features, (b) calculated by averaging image feature similarity values across compounds, (c) calculated by averaging fingerprint feature

similarity values across compounds, and (d) calculated by averaging multimodal feature similarity values across compounds. The labels for x-axis and y-axis of the first matrix are cell image test samples, and the labels for x-axis and y-axis of the later three matrices are compounds.
